# Prospects for Optogenetic Augmentation of Brain Function

**DOI:** 10.3389/fnsys.2015.00157

**Published:** 2015-11-23

**Authors:** Sarah Jarvis, Simon R. Schultz

**Affiliations:** Centre for Neurotechnology and Department of Bioengineering, Imperial College LondonLondon, UK

**Keywords:** optogenetics, neural augmentation, neural coding and decoding, neural engineering, neural modulation, neural prosthetics, BMI (brain machine interface)

## Abstract

The ability to optically control neural activity opens up possibilities for the restoration of normal function following neurological disorders. The temporal precision, spatial resolution, and neuronal specificity that optogenetics offers is unequalled by other available methods, so will it be suitable for not only restoring but also extending brain function? As the first demonstrations of optically “implanted” novel memories emerge, we examine the suitability of optogenetics as a technique for extending neural function. While optogenetics is an effective tool for altering neural activity, the largest impediment for optogenetics in neural augmentation is our systems level understanding of brain function. Furthermore, a number of clinical limitations currently remain as substantial hurdles for the applications proposed. While neurotechnologies for treating brain disorders and interfacing with prosthetics have advanced rapidly in the past few years, partially addressing some of these critical problems, optogenetics is not yet suitable for use in humans. Instead we conclude that for the immediate future, optogenetics is the neurological equivalent of the 3D printer: its flexibility providing an ideal tool for testing and prototyping solutions for treating brain disorders and augmenting brain function.

## 1. Introduction

Combining genetic targeting with optical excitation, optogenetics offers the ability to not only record the activity of large populations of neurons but also manipulate the activity of individual cells. Recording neural activity is achieved by selective expression of activity sensitive fluorophores (such as those from the GCaMP and VSFP protein families, reviewed by Knöpfel, 2012) into neurons, whose activity can then be read out by optical imaging. Manipulation of neural activity can be achieved via the insertion of light-sensitive proteins (opsins) that act as ion channels or pumps into a neuron's membrane and are preferentially controlled by photons of different wavelengths, providing temporal control on the order of milliseconds. Together, this cell type specificity and temporal control results in a tool that can perturb neural circuits with high precision. Since its first application to neural populations in 2005 (Boyden et al., 2005), it has already had substantial impact as a popular technique within the neuroscientific toolkit.

In addition to its use as a research tool, optogenetic stimulation has been suggested as a new approach for neuroprosthetics and treatment of brain disorders. While therapies in these domains have traditionally used electrical or pharmacological techniques, optogenetics has one particular advantage over electrical stimulation in being able to target specific cell classes through gene expression. As a result, specific neuronal populations can in principle be controlled without potential brain-wide, side effects. Likewise, the extremely fine temporal precision it offers on the scale of milliseconds, due to its optical activation, has the advantage over pharmacology of not only acting immediately but also having no washout time. Together, these characteristics have led to optogenetics to be proposed as a viable approach for improved deep brain stimulation (Kravitz et al., 2010), reinstatement of functionality following spinal cord injury (Alilain et al., 2008) and retinal prostheses (Degenaar et al., 2009; Busskamp et al., 2012), amongst other potential clinical applications in humans, following studies using mice, rats and non-human primates as animal models.

The blurred boundary between restoring function and functional enhancement is present for any biomedical intervention. From improving existing function to the incorporation of new streams of information, augmentation of the central nervous system raises specific challenges, from technical issues that are shared with the development of neural therapies, to the more fundamental difficulty of identifying where and how to best modify existing activity to move to the new neural trajectory. In addition, the ethics of the benefits and unintended consequences of intervening in the brain are substantial, as discussed in a recent report by the Nuffield Council on Bioethics 2013.

Does the usefulness of optogenetics as a tool for probing neural circuits automatically translate to its use for neuroaugmentation? Or instead, is it at best limited to the rapid prototyping of novel approaches for enhancing neural processing, much like 3D printers have accelerated for the development of biomedical devices such as prosthetics (including ears, prosthetics and sockets), with the benefit of allowing easy customization? In this article, we examine how well suited is it as a tool for improving, and not merely probing, neural function. By identifying the advantages optogenetics offers over traditional tools for treatment of dysfunction, as well as the hurdles facing neuroaugmentation, we evaluate the use of optogenetics as a practical tool for neural enhancement.

## 2. Optogenetic treatment and neuroprosthetics

A long-held goal of neuroscience has been to identify the specific roles that various neuronal populations play in neural information processing, in order to develop novel therapeutic approaches for brain disorders. The development of light sensitive tools, including opsins and optically activated G protein–coupled receptors (GPCRs), has provided an exceptional tool with which to dissect out the roles of neuronal populations. Critically, their specificity to target neurons by neuronal class and location is a unique advantage, and the ability to test without inducing irreversible changes allows confirmation without long-term damage. In rodent models, optogenetics has been used for investigating disorders such as Parkinson's Disease (Gradinaru et al., 2010), drug addiction (Witten et al., 2010), epilepsy (Bernstein and Boyden, 2011; Tye and Deisseroth, 2012), post-traumatic stress disorder (Sparta et al., 2013) and obesity (Krashes and Kravitz, 2014), among others. Subsequently, refining our understanding of neuronal processing has led to improving treatments by either refining the stimulation protocol or identifying a different target population.

The potential of optogenetics as a more finely targeted alternative to traditional neuromodulatory treatments, such as deep brain stimulation (DBS), has led to the development of optical DBS in rodent models (Aravanis et al., 2007) and proposals for its use in primates (Han and Boyden, 2007; Han, 2012). More recently, combining optical DBS with online monitoring of state has been possible: By integrating optogenetics with fMRI (Lee et al., 2010; Kahn et al., 2013), thus providing the critical link for evaluating the efficacy of the intervention. Specifically, this has been utilized to evaluate the use of optogenetics for the effective control of epileptic seizures, thus allowing the development of less disruptive interventions for temporal lobe epilepsy than are currently clinically available (Armstrong et al., 2013; Krook-Magnuson et al., 2013).

The ability to either increase or decrease activity is an essential aspect of making defined manipulations of targeted elements of the cortical circuit. However, identifying the optimal opsin for a given effect and target neuronal population is challenging. This is illustrated by the recent history of the development of optogenetic retinal prostheses. Originally, channelrhodopsin-2, an excitatory opsin, was proposed to be used to replace function in the retinal ganglion cells layers in conditions such as Retinitis Pigmentosa (RP) and Macular Degeneration (MD). Despite initially promising reports (reviewed in Busskamp et al., 2012), a later study concluded that channelrhodopsin-2 (ChR2) lacks the necessary sensitivity to make its use in a retinal prosthesis viable (Lagali et al., 2008). Soon after, halorhodopsin, an inhibitory opsin, was instead targeted to the photoreceptors. By changing the targeted circuit element, and sign of the perturbation applied, it was possible to significantly improve performance (Busskamp et al., 2010). Given that the retinal circuit has been mapped and extensively studied, this highlights the difficulty in identifying the optimal population to be targeted for even relatively simple networks.

The prospect of viable optically targeted treatments has been further advanced by the development of opsins that have effects beyond their immediate photoactivation, such as step-function opsins (SFO; Berndt et al., 2009) and stabilized step-function opsins (SSFO; Yizhar et al., 2011) that are able to sustain a photocurrent for longer durations (on the order of 30 min) before deactivation via illumination at another wavelength. These opsins thus offer the potential to alter the balance between excitation and inhibition over long-time scales. By doing so over large cortical areas, they offer the possibility to modulate activity which make them suited for treating conditions that are characterized gain dysfunction, such as depression, anxiety, autism, schizophrenia and attention deficit disorders (Yizhar et al., 2011), while minimizing the need for sustained activation. Independently of other clinical hurdles, the reality of this approach hinges on the stability of SFO and SSFOs to sustain a photocurrent over far longer durations, which drastically limits its potential as a therapy in itself. However, a substantial advantage of these opsins is that the strength of their modulation is dependent on the irradiance, which could provide a effective method by which to evaluate the magnitude of the shift required to restore healthy neural functioning, thus indirectly assisting in the development of alternative therapies.

A further use of optogenetics exploits its ability for altering neural activity with high temporal precision. By using short, well-timed pulses, it is possible to not only induce short-term plasticity but also induce long-term potentiation and/or depression (Zhang and Oertner, 2006). This suggests the possibility of reprogramming circuits by either strengthening or weakening connections (Gu and Yakel, 2011; Larsen et al., 2014). The same technique has also been applied to promote regrowth following peripheral nerve injury (Li et al., 2011).

Together, these studies demonstrate that optogenetic technology has many useful properties, including specificity for targeting neuronal populations, activation flexibility due to the range of opsins available, and excellent spatiotemporal control. Given this, we now examine how well suited optogenetics is for augmenting brain function.

## 3. Extending neural flow of information through optical control

Neural augmentation technologies aim to enhance the cognitive capacity or sensorimotor function of the brain. The underlying principles here are conceptually similar to those of neuroprosthetics, in that they both involve altering neural activity or neural circuits in order to redefine input-output relationships. However, there is a fundamental difference. Replacing functionality requires only a crude approximation of absent activity, while enhancement can involve either refining existing activity whilst preserving the brain's ability to process existing signals, or alternatively incorporating new streams of information, thus allowing a neuron to sample input from additional stimuli. The difficulty in interfacing with the CNS whilst preserving natural activity can be observed even at the single cell level. In optogenetics, using high levels of illumination lead to optical initiation of an action potential that effectively overrides the neuron's behavior, so that it produces a spike irregardless of its inputs. A subtler approach to integrating additional signals into the nervous system might instead superimpose such signals onto the existing inputs to the neuron, thus allowing the addition of new information streams into neural circuits without necessarily disrupting the processing of existing streams.

The obstacle of effectively blending information in the brain is well-illustrated in the history of the visual prosthesis. Following earlier observations that applying electrical current to the occipital lobe resulted in the perception of phosphenes (Foerster, 1929), the first visual prosthesis was implanted into the cortex (Brindley and Lewin, 1968), preceding the first retinal prosthesis by nearly a decade (Dawson and Radtke, 1977). Despite improvements to electrode design in the decades since, such as increasing the density of cortical electrodes, as well as an ever-increasing understanding of the visual system, cortical visual prostheses have not yet matched the clinical success of retinal prostheses (Eiber et al., 2013), highlighting the difficulties of artificially altering neural activity within the brain. This is despite the presence of organizational features of the visual cortical areas, such as the retinotopic map, providing a topography for mapping information onto the surface of the cortex which could be coopted for brain-machine interfacing purposes. As one progresses synaptically further from the retina, visual information is organized along more complex—and generally less well understood—dimensions. Consequently, the lack of a well-understood topographic map makes neural interfacing with association areas more difficult. This is even more true of higher cortical areas, such as the prefrontal cortex, which receive information from multiple areas. Thus, although information relating to our interaction with the world may be mapped in some kind of ordered fashion onto its surface, our understanding of that map is incomplete and we are therefore currently unable to exploit it for brain augmentation.

**Figure 1:**
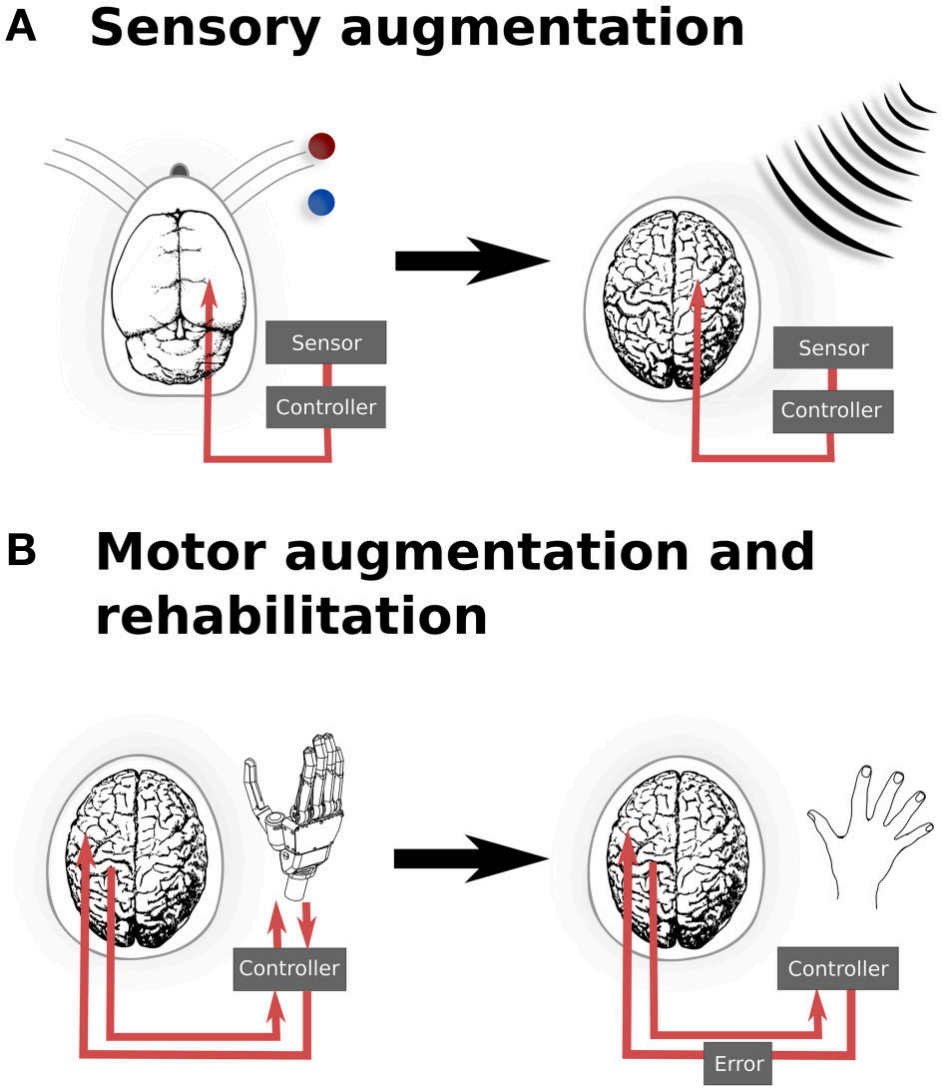
**Translation from experimental and existing therapeutics to clinical applications of neural augmentation using optogenetics**. **(A)** Manipulation of somatosensory codes has been achieved in mouse barrel cortex, whereby optogenetically perturbing the sensory code caused the mouse to incorrectly report the location of the real bar as being in the blue position when the correct location was the red position (O'Connor et al., 2013). In a similar manner, directly interfacing into human somatosensory when an external sensor detects signals that are imperceptible for humans, such as electromagnetic radiation, may allow for additional sensory capabilities. **(B)** Currently, BMI applications only offer the possibility of open-loop applications, by reading neural activity to drive interfaces. Optogenetics could potentially extend BMI to provide currently sensory feedback from the prosthetic back, thus closing the feedback loop (left). This could be extended to refine motor control (right), by supplying an additional sensory error which has been proposed to improve motor rehabilitation following stroke (Wei et al., 2005; Celik et al., 2009). Together, these applications highlight the potential to develop applications from both animal models as well as existing applications, such as BMI. Images: prosthetic hand taken from Patent US Patent App. 10/488,008. Human brain (Wikimedia Commons); Mouse brain (Green, 1966).

However, there is evidence demonstrating that optogenetic manipulation for augmenting simple stimuli is possible. In a recent study, mice were trained to discriminate the location of a bar during whisking (O'Connor et al., 2013). After identifying the coding scheme used within the barrel cortex to encode the location of the bar stimulus, optogenetic perturbations were then applied that would encode, if successful, for the other potential position. The mice reported the bar in the incorrect (virtual) location. While this study was important for the insight it provided into the mechanisms of whisker stimulus encoding, it also provides us with a glimpse into the possibilities provided by illusionary or virtual stimuli. If it is possible to optically signal a false bar position during whisking, then it also hints at the potential of refining sensory inputs based upon additional, non-biological sensors—or potentially even of integrating entirely new sensory inputs into conscious awareness. For instance, it may be possible to augment conscious perception with input from non-visible parts of the electromagnetic spectrum, in order to provide additional sensory capability for working in dangerous environments (Figure 1A).

Augmenting sensory function supplies additional input to the relevant sensory cortex and require a detailed topological map of the neural organization. Similarly, motor outputs can be remapped onto robotic manipulators, allowing augmented motor functionality for external actuators. Neuroprostheses for augmenting motor functionality might use open-loop systems, requiring only the reading of signals from motor cortex. However, closed loop neural interfaces have proved to be important for making BMI a completely integrated replacement for motor function (Donoghue, 2002). In the context of optogenetic brain augmentation, this might involve a fully optical system (e.g., reading out signals from motor cortex using optical imaging of a genetically encodable calcium indicator, together with writing feedback signals into, for instance, somatosensory cortex using optical stimulation of opsins). This potentially also opens up the opportunity to improve learning by augmenting the trajectory error, which provides vital feedback during normal motor learning tasks (Figure 1B). Studies have shown that for gross movements, both error amplification and offsetting improve the amount and speed of adaptation during motor learning, using protocols normally targeted at motor rehabilitation following stroke (Wei et al., 2005; Celik et al., 2009). While these studies have been limited to error augmentation to gross movements, the spatial specificity of optogenetics when applied to topological maps presents the possibility for artificially inducing errors in either a motor-related cortices, to induce a trajectory bias (Wei et al., 2005), or with more difficulty, to generate errors related to subject's knowledge of the current state (Celik et al., 2009).

One requirement for a closed-loop system is the need for optimizing information transfer rates. Motivated by the need for more efficient methods for patients with neuromuscular disorders, much effort has been dedicated to developing classification schemas to optimize information transfer rates (Wolpaw et al., 2002; McFarland et al., 2003). Many BMIs currently use EEG, which is slower and population based, or implanted cortical electrodes, which improve on temporal and spatial resolution for decoding neural activity but lack encoding specificity, particularly for neural stimulation. By replacing this with optogenetics, it becomes possible to record signals with higher temporal and spatial resolution, thereby increasing accuracy, as well as stimulating defined cell types and patterns. This offers the potential of precise decoding of the activity of neural ensembles, which has implications beyond clinical BMI applications. In principle, the provision of a high bit-rate bidirectional interface between the brain and a computer would enable additional computational operations to be outsourced to a computer. This includes, for instance, possibilities ranging from decoding encrypted content using visualized passcodes, through to the external storage of memories (Berger et al., 2011). However, the development of such applications requires human subjects, and is thus unlikely to achieve ethical approval for these applications alone. Yet, as with electrode-based interfaces, such applications of optogenetic brain-machine interfaces may emerge as a by-product of clinical research.

In addition to memory, there is already some evidence that optogenetics can successfully alter cognitive and behavioral processing. In a recent study, it was possible to induce a negative behavior similar to obsessive-compulsive disorder (OCD) by targeting cortico-striatal projections, thus inducing behavior directly (Ahmari et al., 2013). The aim of the study was to test whether hyperstimulation of this pathway in mice would result in OCD-like behavior, which is unable to be tested clinically, but their success demonstrates that optogenetic intervention can change behavioral characteristics—with changes lasting up to weeks after the experiment ended. Similarly, another recent report investigated the role of serotonergic neurons within the dorsal raphe nucleus, which were already known to be involved in signalling reward. Using optogenetics, Miyazaki et al. (2014) established that mice extended their waiting time with a probability that inversely correlated with the delay before serotonergic neurons were optogenetically activated, leading them to conclude that precisely timed stimulation of serotonin neuron correlates with the willingness to wait—a quality they referred to as “patience.”

From sensory and motor augmentation, through to modification of cognitive and behavioral traits, optogenetics uniquely has the specificity and precision to affect the neural correlates of these processes and to improve them. Yet for all the promise, this is still hampered by a common limitation: a deep understanding of the topographic mapping of information onto the relevant cortical areas and of the fine-grained neural coding of cognitive signals. Considerable progress in optogenetic augmentation of cognitive capacity will therefore have to wait for a more detailed understanding of “the cognitive neural code” before augmented neural function becomes an achievable reality.

## 4. Clinical challenges for optogenetic augmentation

Despite the very clear advantages that optogenetics offers for controlling neural activity, there are also four technical hurdles that exist before its translation to applied clinical use. These are opsin delivery, opsin choice, illumination strategies and optical actuators. Additionally, the ethical and regulatory hurdles raised by augmentation using optogenetics are manifold.

To date, the majority of optogenetic studies use mice, due to the large number of transgenic murine strains available for cellular targeting (e.g., using Cre recombinase, Madisen et al., 2012, and now intersectional genetic targeting approaches, Madisen et al., 2015). Cre recombinase targeting is however inapplicable in humans, thus an alternative method must be used. The most likely remaining candidate for opsin delivery is viral transfection, which uses viruses as carriers, such as adeno-associated viruses (AAV) or lentiviruses. Recent studies have confirmed that opsin delivery via AAV can be performed in species other than mice, including rats (Bass et al., 2010; Witten et al., 2010; Stefanik et al., 2013) and primates (Han, 2012). Furthermore, AAVs have been approved by the FDA for use in clinical trials (1995 for the first application; 2005 for the first application within the brain; Carter, 2005), thus removing a key hurdle for the use of optogenetics in clinical trials. An alternative strategy uses transplantable cells deliver opsins to marked sites (Weick et al., 2010); although this approach can suffer from extended opsin expression time, it raises the intriguing prospect of incorporating optogenetic addressability into new tissue formed by stem cell approaches to brain repair.

Different opsin variants have been shown to modify neuronal firing patterns in different ways. The insertion of CatCh, an excitatory opsin, prevented spikes following an initial volley spiking in fast-spiking cells, while the same cell class demonstrated sustained spiking when another actuating opsin, channelrhodopsin fast receiver (FR), was used instead (Mattis et al., 2012). The improvement of opsin design is ongoing, aiming for faster kinetics, preferred excitation wavelength, increased sensitivity and faster recovery (Lin, 2011). Among the newer generation of opsins are Chronos, a improved channelrhodopsin (Klapoetke et al., 2014), and JAWS, a red-shifted light-driven chloride pump (Chuong et al., 2014). As exhaustively testing all combinations of opsins and neuronal populations is impossible, this issue highlights the importance of good opsin characterization and models for determining suitable opsin-neuron combinations within any given neural circuit. In this, the development of accurate computational models of neurons, circuits (Potjans and Diesmann, 2014) and opsins (Nikolic et al., 2009; Grossman et al., 2013; Nikolic et al., 2013) will be vital for refining the matching of opsin to neuronal population and illumination protocols (Lin, 2011; Mattis et al., 2012; Jarvis et al., 2014).

A more practical concern is the light source, which can either be externally located or implantable. Similarly to the limitations that have troubled other biomedical devices, such as electrical DBS or retinal implants, both external and implantable devices have limitations: respectively, these are the need to open a pathway through the skin, and the need to provide power, either by an implanted battery or by transcutaneous transmission (O'Handley et al., 2008). Further practical limitations apply due to the need to deliver light to structures that can be located deep within the brain. Light penetration through tissue is limited by scattering. This is already limiting in mice (although new redshifted opsins such as ReaChR Lin et al., 2013 and JAWS Chuong et al., 2014, are increasing the achievable penetration depth). This factor becomes a major hurdle upon scaling up to human subjects. One approach to the solution of this problem is to target deep brain structures using penetrating optrodes, analogous to the use of penetrating electrodes in DBS. An additional issue that may arise in larger animals is to ensure the uniform spread of light over a wide area of tissue, i.e., ensuring that the falloff rate for illumination is sufficiently low such that the entirety of targeted areas are illuminated, whilst avoiding tissue damage due to overheating (Han, 2012).

Additionally, it is currently unclear that actuating an entire population of cells will be sufficient to provide useful input to the brain. While population firing rate codes have been demonstrated in some cortical areas (Georgopoulos et al., 1986), even firing rate codes may be “labeled line” codes in which cell identity matters (Montani et al., 2007; Schultz et al., 2009). Processing in some brain circuits has been proposed to occur through precise phase latencies within oscillatory activity (Engel et al., 2001), and the mechanisms underlying coding of cognitive information within the brain are still far from clear. For these latter schemes, whole field illumination of neurons in even a class-specific manner will clearly not be effective. Instead, only a specific subset of cells, such as an orientation column, should be targeted which is currently impossible using whole-field illumination.

**Figure 2:**
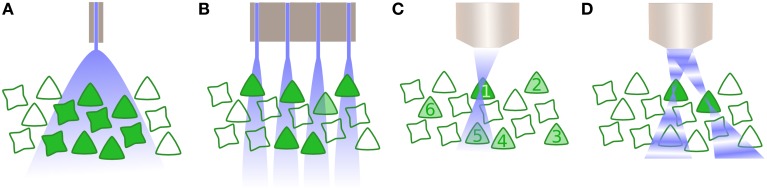
**Activating neurons to drive activity**. **(A)** Whole field illumination via diode, either located at the cortical surface or inserted within the tissue. In both instances, all neurons within the illuminated area are activated. **(B)** An array of photodiodes illuminates subpopulations in the lateral plane, illustrated here in combination with opsin specificity for only one class of neuron. However, targeting is not constrained in the axial dimension and likewise drives multiple neurons within a column corresponding to each individual diode. **(C)** Multiphoton excitation results in a spatially constrained point spread function (PSF) in lateral and axial planes. Here, the 2P beam is targeted for the first neuron, whose soma is significantly larger than the PSF thus requiring scanning the neuron and increasing the dwell time. Driving other selected neurons within the population (2–6) is performed sequentially. **(D)** Digital holography utilizes wavefront shaping, in which a spatial light modulator alters the phase of beams in order to create multiple PSFs, allowing multiple neurons to be simultaneously but independently driven.

This leads directly on to the last significant technical hurdle for clinical optogenetics: how can a large number of neurons within a population be driven with precise spatial, and defined temporal, resolution? The majority of optogenetic experiments to date have employed single photon excitation of opsins, typically supplied by a light emitting diode or fibre-coupled laser. The disadvantage with such approaches is that optogenetic activation is then not spatially limited, and consequently all opsin-expressing cells within an area of tissue are affected (Figure 2A). For some optogenetic applications, this may not be sufficient. Instead, it is critical to target subpopulations of neurons, then alternatives are available, such as the use of techniques such as multi-site light emitting diode arrays (Grossman et al., 2010). However, such approaches are in practice limited to the retina or *in vitro* preparations, as they provide negligible to no confinement within the axial plane (Figure 2B).

Two further possibilities exist that exploit optical technology to provide precise spatiotemporal patterning. The first is multiphoton excitation (Rickgauer and Tank, 2009), typically 2-photon (2P), which is spatially confined enough to allow the optical activation of individual cells. By using the interaction of two photons of longer wavelengths, which have less scatter, a small point spread function (PSF) is created that is constrained in the axial as well as lateral dimension (Figure 2C) and can be relocated in space, thus activating individual neurons throughout the tissue volume. However, as the PSF is typically smaller than the soma, 2P excitation is insufficient to initiate an action potential without scanning within the cell—which in turn reduces the temporal resolution of the technique (Rickgauer and Tank, 2009). Another recent approach uses wavefront shaping methods, such as digital holography or generalized phase contrast (Figure 2D), and by manipulating the phase of light with a spatial light modulator, create a defined spatial pattern of light that can similarly scan multiple neurons in a volume of tissue (Papagiakoumou et al., 2010; Oron et al., 2012). Both multiphoton excitation and wavefront shaping are limited at a rate proportional to the population size of the neurons to be targeted. Furthermore, despite being tested *in vivo* as well as *in vitro*, they only exist for head-fixed subjects, thus currently preventing its application for experiments including freely behaving subjects. Yet, as precise spatiotemporal patterning is likely to be necessary to take advantage of the full capabilities of optogenetic manipulation of brain circuitry, particularly where synaptic plasticity mechanisms are involved, this area of technological development is one to watch closely.

In addition to technical hurdles, the regulatory requirements for bringing optogenetics to clinical reality are also substantial. As discussed, the FDA has already cleared the use of AAV-delivered, providing an option for opsin delivery (Carter, 2005). However, opsin expression application in human, in which light-sensitive proteins are inserted into cellular membrane via genetic manipulation, has not been cleared and would likely require similar clearance to other gene therapies. This is compounded with the difficulty in determining the correct levels of opsin expression, as overexpression has been demonstrated to have cytotoxic effects. The FDA has recently cleared an application for optogenetic gene therapy for the treatment of RP, which will allow clinical trials in humans to commence (Francis et al., 2013; RetroSense, 2015). The treatment aims to restore photosensitivity of photoreceptors, bypassing the need for a separate light activation source. This is in contrast to cortical optogenetic application which would additionally require implanted optrodes, placing them in the highest band for both the FDA and EU regulatory approval. In addition to the usual considerations associated with implantable device, implanted optical devices have an additional constraint of minimization of energy via heat lost to prevent tissue damage. Determining the distribution of energy by optical delivery devices has been modeled (Ozden et al., 2013), however althought the FDA have determined power limits for MRI that allow temperature changes of 1°*C*, there are, as yet, no limits for power limits for chronically implanted optrodes (Ozden et al., 2013). For chronic usage, this may require significantly lower limits for power, thus restricting the range of illumination.

Finally, the ethical considerations that surround neuroenhancement are substantial and have been discussed elsewhere, including in this issue (Clark, 2014; Shook et al., 2014). Many of the same arguments for enhancement of cognitive abilities via pharamacological (Hyman, 2011), transcranial or electrical means are similarly applicable here for optogenetics: the development of a cognitive “arms race"; the question of who is in control of the augmenting device; and safety, both due to unintended consequences of manipulating neural activity as well as from the treament itself. The latter is particularly pertinent for the application of optogenetics, as it is highly invasive, requiring both manipulation of genetic material as well as the subcranial placement of devices to provide optical activation. This raises the ethical cost of optogenetics, such that its capacity for neuroenhancement must be substantially higher in comparison to other treatments to warrant its use preferentially. However, this provides an ethical impasse: how should these critera be tested and developed for optogenetics, while the risk and invasiveness of clinical optogenetics remains high? It may well be that such clinical trials may emerge as a by-product of optogenetic therapies for treatment of existing dysfunction or BMIs, rather than augmentation; or instead that it will ultimately require the use of similar but separate therapies, such as targeted nanoparticles (Carvalho-de Souza et al., 2015) which have the same advantages as optogenetics, but with diminished risks, to make neuroenhancement not only ethically more attractive but also clinically attainable.

## 5. Conclusion

Optogenetics has opened up a variety of new experimental paradigms in neuroscience. Its key advantage lies in the ability to target neuronal populations with precise spatiotemporal activation with immediate and reversible effect, which has been advantageous for untangling the contribution of neuronal populations in neural processing in both anatomical and behavioral studies (Tye and Deisseroth, 2012). These advantages also place optogenetics as a well-suited tool to assist in the *development* of neurological therapies and, correspondingly, neural enhancements.

The application of optogenetics as a tool for *direct use* in neural modulation itself is less certain. The technological development of optogenetics is still in progress, with opsin molecular engineering, opsin delivery and optical stimulation techniques advancing rapidly, which will undoubtedly increase the precision of optogenetics and with it, our ability to manipulate neural circuits. However, the translation of optogenetics from research tool to clinical application has additional stipulations, particularly for its use in humans. From the challenges of opsin delivery to the difficulties in optically driving neurons with implantable devices, applying optogenetics outside of research remains a remote possibility in the foreseeable future. Yet in themselves, these elements are not the most significant constraint on the application of optogenetics for neural augmentation, which is instead our understanding of the neural codes that we are attempting to adapt. In the meantime, optogenetics offers a viable possibility for the development of novel neural therapies, by providing a robust capability to technically refine stimulation protocols and evaluate the effect of modulating activity levels of different neuronal populations.

### Conflict of interest statement

The authors declare that the research was conducted in the absence of any commercial or financial relationships that could be construed as a potential conflict of interest.
